# Tailoring hierarchical ZSM-5 with water-soluble polymers templates for BTX production in methanol-to-aromatics

**DOI:** 10.1039/d5ra08403b

**Published:** 2026-02-04

**Authors:** Arash Abbasi, Kobra Pourabdollah, Babak Mokhtarani

**Affiliations:** a Chemistry and Chemical Engineering Research Center of Iran Tehran Iran pourabdollah@ccerci.ac.ir

## Abstract

Hierarchical ZSM-5 zeolites were synthesized by one-pot methods utilizing water-soluble polymers—polyvinylpyrrolidone (PVP) and polyethylene glycol (PEG)—and were compared with a desilicated (DS/ZC) typical microporous ZSM-5 (ZC). Thorough evaluation utilizing XRD, FT-IR, NH_3_ physisorption, SEM and TEM, ICP-OES, NH_3_-TPD, and TGA demonstrated that PVP-templated ZSM-5 (PVP/ZC) displayed distinct mesoporosity while maintaining its inherent microporous structure, resulting in a balanced allocation of weak and strong acid sites. All catalysts were assessed in the methanol-to-aromatics (MTA) reaction at 400 °C, with a weight hourly space velocity (WHSV) of 5 h^−1^ and atmospheric pressure (time on stream = 3 h), resulting in over 99.5% methanol conversion. PVP/ZC attained the highest BTX selectivity of 51.3% and an overall aromatic selectivity of 60.1%, due to its uniform mesoporosity, reduced diffusion pathways along the *b*-axis, and optimized distribution of acid sites, which collectively minimized secondary C_9+_ formation (8.8%) and coke deposition (3.27 wt%). The findings indicate that water-soluble polymer templates, specifically PVP, facilitate the customized synthesis of hierarchical ZSM-5 catalysts, which improve BTX production and stability in the MTA process, providing a cost-effective and environmentally friendly alternative to conventional templating and post-synthetic treatments.

## Abstract 2

Hierarchical ZSM-5 catalysts were synthesized *via* a one-pot, eco-friendly method using water-soluble polymer templates with high crystallinity and uniform mesoporosity in the MTA reaction.

## Graphical abstract

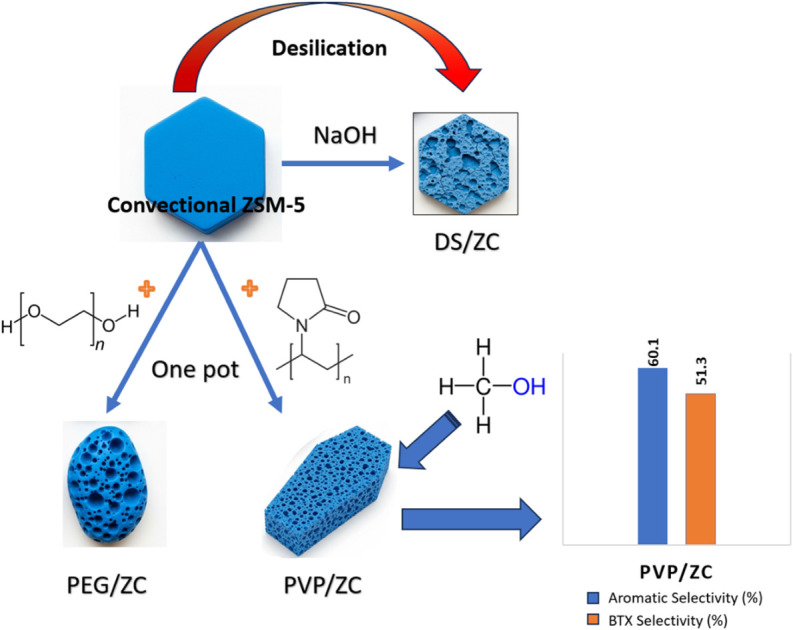

## 1. Introduction

BTX stands for benzene, toluene, and xylene, which are essential aromatic hydrocarbons in the petrochemical industry. These compounds serve as key building blocks for medicines, explosives, perfumes, agrochemicals, polymers, resins, fibers, rubbers, and detergents, making them widely used synthetic monomers and solvents. Owing to their stable reactive ring structures, BTX compounds play a crucial role in the large-scale production of value-added chemicals.^1–3^ By 2025, the global aromatics market is predicted to be worth USD 290.12 billion, this means a Compound Annual Growth Rate (CAGR) of more than 5.67% from 2020 to 2025.^4^ Traditionally, the production of aromatics has relied on petroleum-derived processes, such as naphtha reforming and steam cracking.^5,6^

Many methods and sources have been investigated for the synthesis of aromatic hydrocarbons. These include biomass conversion to aromatics,^7,8^ the conversion of alcohols—more especially, methanol and ethanol—into aromatics,^9–11^ the pyrolysis of waste polymers to aromatics,^12–14^ and the use of lignin as a precursor in aromatic production.^15,16^ Among these approaches, the methanol-to-aromatics (MTA) process has attracted significant attention due to its high potential for efficient aromatic production. Methanol is considered an excellent C_1_ building block because of its versatility: it can be derived from diverse carbon sources such as biomass gasification, coal gasification, natural gas reforming, municipal waste, and even CO_2_ hydrogenation. This flexibility enables the production of low-carbon, and in some cases even carbon-negative, aromatics.^9,17,18^

HZSM-5 zeolite has a special three-dimensional net structure that is specifically targeted towards aromatics. It is typically used in MTA and exhibits superior catalytic activity.^19–21^ ZSM-5 consists of crystalline aluminosilicates with uniform micropores smaller than 2 nm. Its channel structure contains 10-ring sinusoidal channels (0.55 nm × 0.51 nm) intersecting the parallel 10-ring channels (0.56 nm × 0.53 nm).^22^ However, because ZSM-5 has narrow channels, macromolecules and their products can't diffuse freely, so they spend more time inside the channels. As a result of this, coke can easily block those narrow pores, thus leading to catalyst deactivation.^23,24^

To overcome the limitations of conventional ZSM-5, researchers have developed various strategies to enhance its diffusion properties and, consequently, its catalytic performance.^24^ These approaches include the synthesis of hierarchical or mesoporous ZSM-5 zeolites, as well as the preparation of small-crystallite or nanosized forms.^25^ Hierarchical micro–mesoporous ZSM-5 is particularly attractive for industrial applications because it combines the advantages of both microporous zeolites and mesoporous materials. In ZSM-5, micropores provide strong shape selectivity for target products, while mesopores facilitate the diffusion of larger molecules and increase the catalyst's tolerance to coke formation.^26^ In addition, mesopores significantly improve the accessibility of acid sites located within the narrow channels of the zeolite. This structure enables reactants to reach active sites more readily, enhances catalytic efficiency by shortening diffusion pathways, and allows products to exit the pores quickly, thereby reducing secondary reactions and improving selectivity toward desired products.^25,26^

There are two main ways to make hierarchical zeolites: *in situ* procedures and post-synthetic techniques, which are also called “bottom–up” and “top–down” strategies.^27,28^

Desilication is typically performed by treating the zeolite with an alkaline solution, whereby framework silicon is selectively removed to generate secondary porosity,^29–31^ dealumination, generally performed with an acid solution,^32,33^ and recrystallization.^34,35^ Ma *et al.*^25^ conducted a thorough examination of the post-treatment processes, including dealumination, desilication, surface modification, and their combinations, on ZSM-5 zeolite. They utilized the synthesized zeolites for the MTA process. Following the conventional synthesis method, which produced nano-sized particles, desilication was carried out using NaOH, a combination of NaOH and TPAOH, dealumination with HCl, and surface modification with oxalic acid. Evaluation of the effects of these treatments on the MTA process revealed that the highest aromatic selectivity attained was 55%. Another study showed how alkaline treatment at different levels affected the texture and acidity of ZSM-5 zeolites (Si/Al = 23.7). This confirmed the creation of mesopores and changes in acidity. Compared to moderate alkaline treatment, a 0.4 mol L^−1^ NaOH treatment made more mesopores. However, it also reduced the amount of Brønsted acid sites, resulting in a BTX selectivity of 34.7%.^34^ The post-treatment method is characterized by its simplicity; however, it is accompanied by several significant disadvantages. This approach requires extreme conditions that may lead to environmental pollution, induce defects within zeolites, cause partial structural collapse of the zeolite framework, and result in inadequate control over the selective and precise extraction processes.^35^

According to the bottom–up procedure, two main strategies exist for preparing hierarchical zeolites. The first involves creating intercrystalline mesopores by controlling zeolite crystal growth through inhibitors or optimizing synthesis conditions to promote nucleation by increasing synthesis time^36^ or using zeolite seed-assisted methods,^37^ thereby reducing crystal size. The second strategy introduces meso- or macro-pores by incorporating soft and hard templates during crystal growth, which occupy space and create mesopores.^35,38^ By adjusting the gel composition of the precursors, Shao *et al.*^39^ developed a series of nano-aggregated hierarchical ZSM-5 catalysts. They used these catalysts in the MTA process and showed that the acidity of the ZSM-5 catalysts increases as the particle size goes down, which is linked to the generation of BTX. The maximum selectivity for BTX reported was 39.6%.

Another study demonstrated that a nanocrystalline hierarchical ZSM-5 catalyst combined the advantages of both nanosized and hierarchical structures. This catalyst is synthesized using 3-glycidoxypropyltrimethoxysilane (KH-560) as a soft template. The template produces uniform spherical aggregates with high porosity and tailored acidity. The availability of numerous strong Brønsted acid sites coupled with a good Brønsted to Lewis acid sites ratio highly promotes the aromatization reaction for BTX production, with a yield of 36.5%.^16^ Wang *et al.*^40^ carried out a synthesis of hollow hierarchical ZSM-5 zeolite *via* a multi-step process in a thorough investigation. Initially, they fabricated Zn/mesoporous aluminosilicate spheres (MASS), which served as a foundational precursor. Following this, a composite known as Zn/MASS@silicate-1 was developed with poly(diallyldimethylammonium) (PDDA), which acted as a robust hard template throughout the process. In the final synthesis stage, the *in situ* crystallization of Zn/MASS@silicalite-1 was facilitated through a vapor phase transport (VPT) treatment, using *n*-butyl amine to synthesize the final hollow hierarchical sample.^40^

The resulting ZSM-5 zeolite demonstrated an acidity profile comparable to traditional zeolites. However, a significant advantage emerged from its larger mesoporous volume, which was shown to enhance aromatic selectivity by 31.9%.^40^ While the bottom–up approach has numerous benefits associated with it, it is also riddled with several challenges. The utilization of templates for the mesoporous structures, for instance, organosilanes^41,42^ and surfactants,^43^ or synthesis complex soft templates,^44,45^ may be costly or complicated and introduce concerns about environmental sustainability. Further, the use of multi-step procedures contributes to the complexity and duration of the synthesis process,^40^ which, in effect, restricts the scalability of the catalyst. Additionally, employing structure-free methods and achieving precise tuning of the synthesis conditions can be quite challenging.^46,47^ Researchers have investigated hierarchical ZSM-5 one-pot synthesis with eco-environment-friendly templates to address the challenges mentioned. The utilization of glucose,^48^ cellulose,^49^ amino acids,^50^ and water-soluble polymers^51,52^ as low-cost and green templates for the synthesis of hierarchical ZSM-5 has been thoroughly investigated. Water-soluble polymers like polyethylene glycol (PEG), polyvinylpyrrolidone (PVP), and pluronic F127 have shown remarkable properties for modifying and creating hierarchical ZSM-5 zeolites.

For example, Zhou *et al.*^52^ synthesized one-pot hierarchical lamellar ZSM-5 with PEG-20000 and applied it in methanol-to-propylene (MTP) conversion. PEG was selected for its physical properties and tunable molecular weight, both of which influence nucleation and crystal growth mechanisms. In another study, cyclohexane oxidation was carried out using a one-pot hierarchical ZSM-5 synthesized with PVP. PVP facilitates the formation of a three-dimensional structure due to its good solubility in water. He appropriate amount of PVP is critical, as it stabilizes the framework during the hydrolysis of tetraethyl orthosilicate (TEOS).^51^ As far as our knowledge goes, few studies have been conducted that used a water-soluble polymer to synthesize hierarchical ZSM-5 and employed it for the methanol to aromatic (MTA) process. Tian *et al.*^53^ synthesized hierarchical ZSM-5 zeolites with varying Si/Al ratios using Pluronic F127 and Pluronic P123 as soft template agents through steam-assisted crystallization. The resulting hierarchical ZSM-5 exhibited a larger external surface area, reduced diffusion resistance, and an increased number of acid sites. These characteristics facilitate the aromatization reaction, resulting in enhanced aromatic performance and BTX selectivity, which was approximately 50% greater than that of microporous ZSM-5.

This work reports the synthesis of new hierarchical ZSM-5 zeolites with water-soluble polymeric secondary templates, *i.e.*, PVP and PEG, *via* a cost-effective and green approach. In this synthesis, *n*-butylamine was employed as the primary template, providing a cheaper and greener alternative to tetrapropylammonium hydroxide (TPAOH).

Additionally, hierarchical zeolites were synthesized *via* the desilication method for the purpose of comparative analysis. The resulting zeolite samples were subsequently evaluated in the methanol-to-aromatics (MTA) process, with a focus on investigating the influence of their morphological characteristics and acidity properties on the efficiency of the MTA process.

## 2. Experiment section

### 2.1. Material

The reagents used in the experiment include tetraethyl orthosilicate (TEOS), sodium aluminate (NaAlO_2_), sodium hydroxide (NaOH), *n*-butyl amine (NBA), and polyethylene glycol with a molecular weight of 10 000 (PEG), all of which were purchased from Merck. Polyvinylpyrrolidone K12 with a molecular weight of 3500 (PVP) was obtained from Thermo Scientific Chemicals, and methanol was sourced from Dr Mojallali Industrial Chemical Complex Co. All reagents were utilized without any purification.

### 2.2. Catalyst preparation

In a typical synthesis for microporous ZSM-5, herein referred to as ZC, 0.21 g of NaAlO_2_ and 0.26 g of NaOH were initially dissolved in 48 mL of distilled water at room temperature and agitated for 30 minutes to ensure homogeneity. Subsequently, 2.43 g of NBA was introduced to the mixture, followed by continuous stirring. Afterward, 13.8 g of TEOS was added dropwise, stirring the mix for 90 minutes to yield a clear gel. The final gel composition was Si: 0.02 Al_2_O_3_: 0.5 NBA: 0.1 NaOH: 40 Water.

The resultant gel solution was then transferred to an autoclave, where it was subjected to 180 °C for 48 h to promote the crystallization process. Upon completion of the synthesis, the obtained solid was filtered and rinsed with distilled water until a neutral pH was achieved, dried at 110 °C overnight, and calcined at 550 °C for 5 hours.

The abovementioned method is also used to synthesize hierarchical ZSM-5 with different molar ratios. NaAlO_2_ and NaOH are dissolved in distilled water, and then NBA is added to this mixture and agitated to ensure all substances mix perfectly. Before adding tetraethyl orthosilicate (TEOS), a certain amount of mesopore template, PVP, or PEG, is combined with the solution for 30 minutes before adding TEOS dropwise. The final gel compositions for PVP/ZC and PEG/ZC are Si:0.02 Al_2_O_3_:0.5NBA:0.2Na:60W:0.0066PVP, and Si:0.02 Al_2_O_3_:0.5NBA:0.2Na:60W:0.002PEG, respectively.

For comparison with the one-pot hierarchical ZSM-5 synthesized, DS/ZC was produced by the desilication method. For a typical run, 5 g of ZC catalyst is added to a 0.2 Molar NaOH solution with a 30 : 1 solution/solid ratio at 70 °C for 1 h. The slurry solution is filtered to collect the catalyst, washed with distilled water, dried at 110 °C overnight, and calcined at 550 °C for 5 hours.

To obtain the H-form of the synthesized catalyst, all catalyst samples underwent ion exchange with a 1 molar NH_4_NO_3_ solution in a 20 : 1 solution/solid ratio at 80 °C for 3 h over two consecutive cycles, followed by drying at 110 °C overnight and calcining at 550 °C for 5 hours.

### 2.3. Catalyst characterization

The crystallinity of the synthesized ZSM-5 zeolites was analyzed using powder X-ray diffraction (XRD) patterns obtained from a Philips X'Pert Pro Materials Powder Diffractometer, which was equipped with Cu Kα (*λ* = 1.544 Å) radiation. The 2*θ* range measured was from 5° to 50°.

Functional groups were evaluated within the 400–4000 cm^−1^ range using Fourier Transform Infrared Spectroscopy (FTIR). This assessment was conducted after the sample was blended with KBr and formed into tablets for characterization utilizing the PerkinElmer Spectrum 65 FT IR Spectrometer.

N_2_ adsorption–desorption isotherms were measured using a BELSORP Mini II gas adsorption analyzer. Before the measurements, the zeolite sample was degassed under vacuum at 200 °C for 3 hours. The surface area was determined using the Brunauer–Emmett–Teller (BET) method, while the pore size distribution was obtained through the Barrett–Joyner–Halenda (BJH) method. The total pore volume was calculated based on the amount adsorbed at a relative pressure of 0.99. Additionally, the *t*-plot method was employed to analyze micropore information.

Inductively coupled plasma optical emission spectrometry (ICP-OES) was performed using a Spectro Arcos analyzer to identify the concentration of Si and Al.

The samples' morphologies were analyzed through scanning electron microscopy (SEM) and transmission electron microscopy (TEM). SEM characterization was conducted with a Tescan VEGA series SEM operating at 20 kV. Before analysis, the samples were coated with a gold film (15–20 nm) using Q150 R ES by Quorum Co. For transmission electron microscopy, a Philips EM208S was used to capture TEM images at an accelerating voltage of 100 kV. The sample was dispersed in ethanol before being placed in the TEM instrument for examination.

The concentration of acidic sites was measured using temperature-programmed desorption (TPD) of ammonia, conducted with the NanoSORD Sensiran Co. instrument. The catalyst samples were initially cleaned by flowing N_2_ for 1 hour at 300 °C and were then cooled down to 100 °C. Following this, the samples were saturated with ammonia by exposing them to an atmosphere containing 10% NH_3_ in He for 1 hour. Finally, TPD measurements were performed within a temperature range of 100 to 700 °C at a heating rate of 10 °C min^−1^.

Thermogravimetric analysis (TGA) of the catalysts following the MTA reaction was conducted using a Netzsch TGA 209 F1. The analysis was performed at a ramp rate of 10 °C per minute in an air atmosphere, reaching temperatures up to 900 °C. This analysis aimed to investigate the formation of coke on the catalysts.

### 2.4. Catalytic test

The MTA reaction was conducted in a continuous fixed-bed reactor with an inner diameter of 10 mm and a length of 100 cm. The reactor was filled with 0.6 g of zeolite (20–40 mesh) mixed with 1.8 g of inert sea sand (10–20 mesh). Before the reaction, the catalyst was heat-treated in a nitrogen flow (40 cm^3^ min^−1^) at 450 °C for 90 minutes under atmospheric pressure. After heat treatment, the reactor temperature was lowered to 400 °C, which was controlled by a K-type thermocouple placed at the center of the catalyst bed.

Methanol was then pumped into the reactor using a syringe pump at a liquid weight hourly space velocity (WHSV) of 5 h^−1^, while nitrogen served as a carrier gas at a flow rate of 40 cm^3^ min^−1^. The exit stream from the reactor was directed through a chilled-water condenser to separate it into gas, liquid hydrocarbons, and water fractions. The product was collected and subsequently analyzed utilizing a Varian Star 3800 gas chromatograph, which was outfitted with both a flame ionization detector (FID) and a thermal conductivity detector (TCD). To ensure reproducibility, each catalytic test was performed at least three times under identical conditions, and the variations in conversion and selectivity were within ±2%.

The conversion of methanol and the selectivity of products were calculated using the following formula (eqn (1) and (2)):1
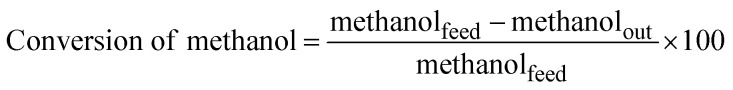
2
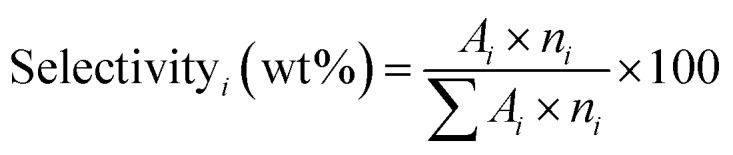
where, *A* represents the peak area, and *n* represents the number of carbon atoms in the product *i*.

The carbon balance was determined from the carbon content of the inlet methanol as well as all gaseous and liquid carbon-containing products. The carbon balance ranged from 97% to 102% across all catalytic runs, which is within the acceptable range for fixed-bed MTA systems.

## 3. Results and discussion

### 3.1. Catalysts characterization

#### 3.1.1. The XRD analysis

**Fig. 1:**
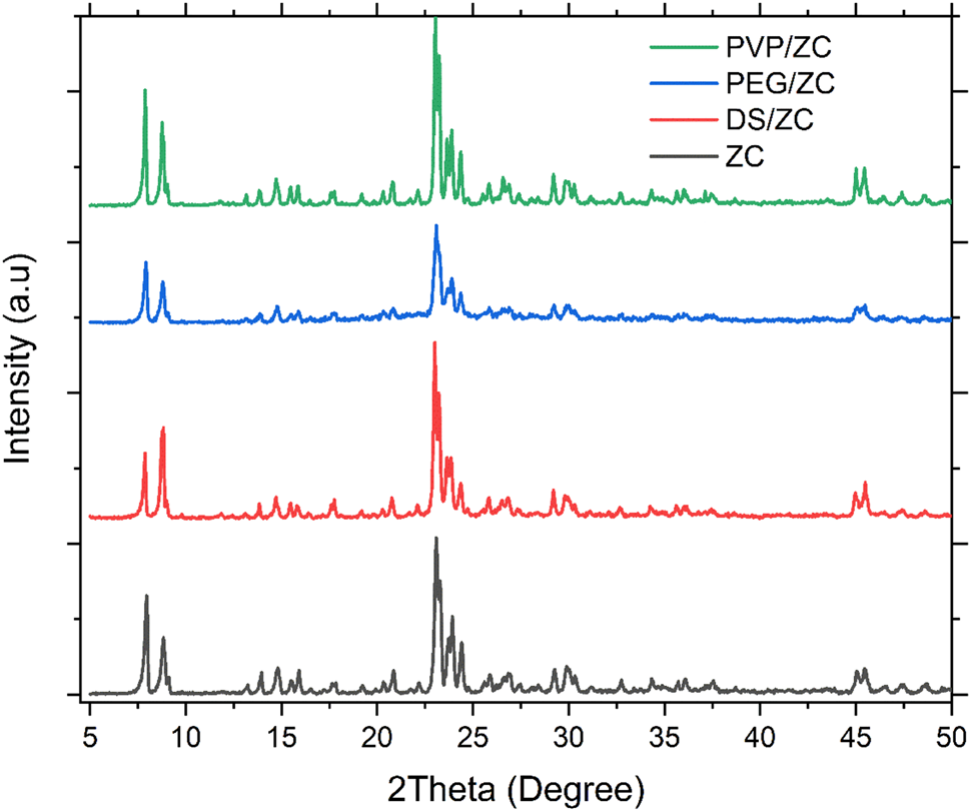
XRD spectra of the four prepared ZSM-5 catalysts.

Fig. 1 shows the XRD spectra of the four prepared ZSM-5 catalysts. All samples exhibit peaks at 2*θ* values of 7.9, 8.8, 23.0, 23.9, and 24.3, which correspond to the (101), (200), (501), (151), and (303) planes of the MFI structure.^54,55^ These characteristics indicate successful synthesis of ZSM-5 zeolites. The degree of crystallinity was calculated from the peak area between 2*θ* = 22–25, using the crystalline ZC sample as a reference, and the results are shown in Table 1. Compared to HZSM-5 (100%), the relative crystallinity of PVP/ZC and DS/ZC is approximately 102% and 96%, respectively. Alkali treatment can induce defects in the zeolitic framework, leading to a reduction in relative crystallinity.^56^ These findings align well with the ICP results presented in Table 1. PEG/ZC exhibits the lowest relative crystallinity, likely due to the presence of PEG. It can promote nucleation by acting as a swelling agent, but it also adsorbs onto the surfaces of the nuclei, inhibiting their growth. As a result, a large number of tiny nuclei are formed that do not develop into well-ordered crystals, leading to an increased amorphous fraction.^57^ This observation is consistent with the SEM image (Fig. 4). PVP/ZC exhibits a relative crystallinity of 102%. These findings indicate that adding PVP may enhance the nucleation and growth of zeolite ZSM-5 crystals.^58^

**Table 1:** Physico–chemical properties of the prepared catalysts

Catalyst	R.C.^a^ (%)	Si/Al^b^	*S*_BET_ c (m^2^ g^−1^)	*S*_Micro_ d (m^2^ g^−1^)	*S*_ext_ e (m^2^ g^−1^)	*V*_Total_ f (cm^3^ g^−1^)	*V*_Micro_ g (cm^3^ g^−1^)	*V*_Meso_ h (cm^3^ g^−1^)
ZC	100	24.1	308.9	255.1	53.8	0.179	0.165	0.014
DS/ZC	96	29.6	373.6	283.2	90.4	0.329	0.207	0.122
PEG/ZC	76	19.7	322.9	203.9	119.0	0.314	0.062	0.252
PVP/ZC	102	21.3	359.1	278.2	80.9	0.287	0.104	0.183

a Relative crystallinity by XRD data.

b Determine by ICP.

c Obtained by the BET method using adsorption data.

d Measured by *t*-plot method.

e (*S*_ext_ = *S*_BET_ − *S*_Micro_).

f Estimated from the adsorbed amount at *p*/*p*_0_ = 0.995.

g Measured by *t*-plot method.

h *V*_Meso_ = *V*_Total_ − *V*_Micro_.

#### 3.1.2. FT-IR analysis

**Fig. 2:**
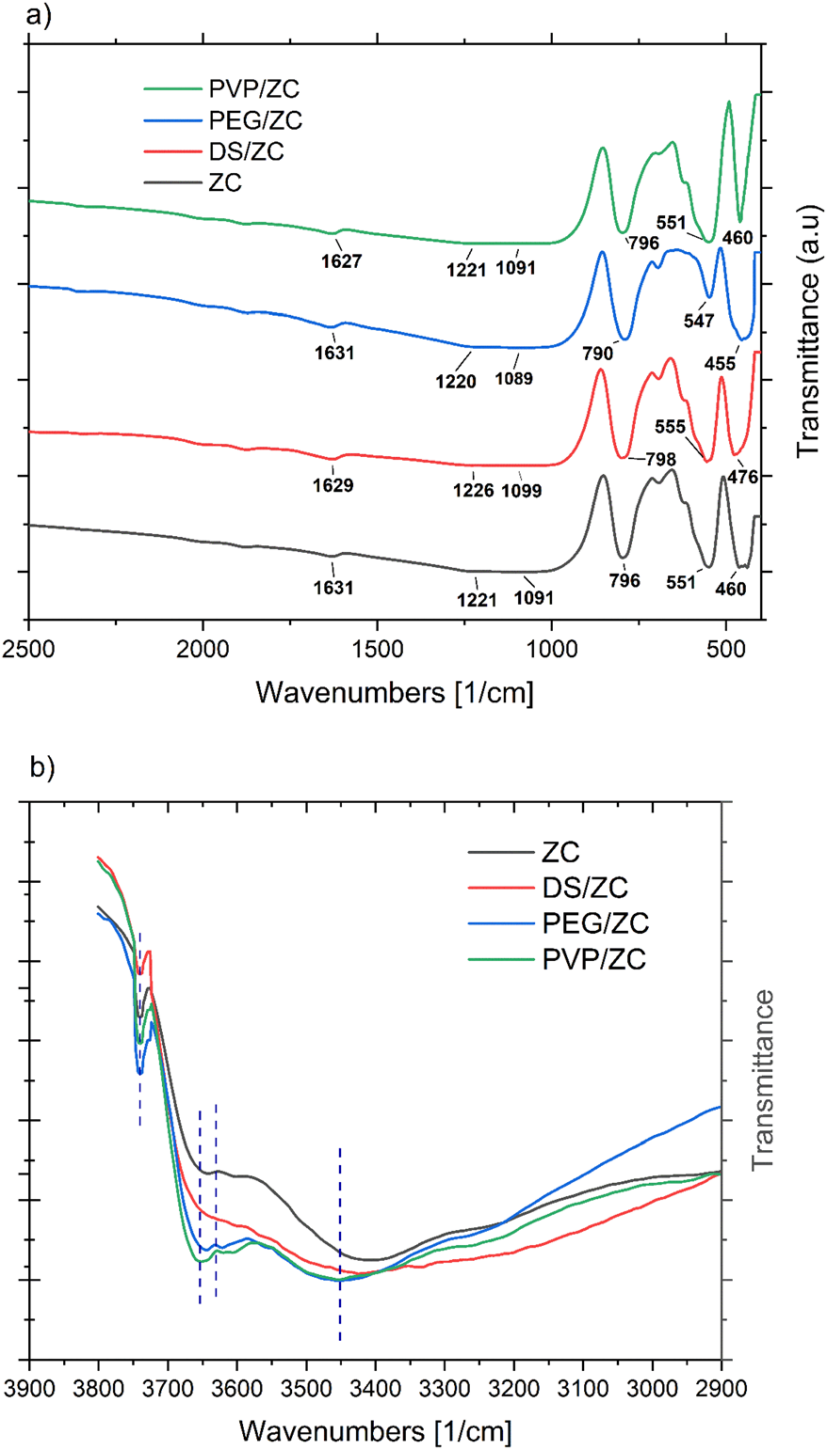
FT-IR spectrum of ZSM-5 adsorbents in the range of 400–2500 cm^−1^. (b) FT-IR spectrum of ZSM-5 adsorbents in the range of 2900–3900 cm^−1^.

The FT-IR spectra of all samples are presented in Fig. 2a and b. All of the as-synthesized samples display characteristic vibrational bands related to the MFI topological structure. The absorption bands at approximately 450–470 cm^−1^ and 550–560 cm^−1^ correspond to the internal framework vibrations and the double five-member ring vibrations, respectively. The peaks at 790–800 cm^−1^, 1050–1100 cm^−1^, and 1220–1230 cm^−1^ are attributed to the symmetric, asymmetric, and external asymmetric T–O–T (T = Al or Si) vibrations, respectively.^38,56^

According to Fig. 2b, the broadband at wavelengths around 3400 cm^−1^ indicates the interaction between silanol groups due to internal defects. Furthermore, the peaks in the range of 3500–3800 cm^−1^ are indicative of the presence of hydroxyl groups, *i.e.*, Al(OH)Si, in zeolite samples. The isolated external silanol is related to the band at 3740 cm^−1^, and that of free internal silanol is related to the band at 3720 cm^−1^; moreover, peaks at 3630–3660 cm^−1^ are ascribed to extra-framework aluminum species or partly hydrolyzed aluminum species.^38,56,59^ These diagnostic absorption peaks are consistent with previously reported FT-IR findings on H-ZSM-5 zeolites.

#### 3.1.3. BET analysis

**Fig. 3:**
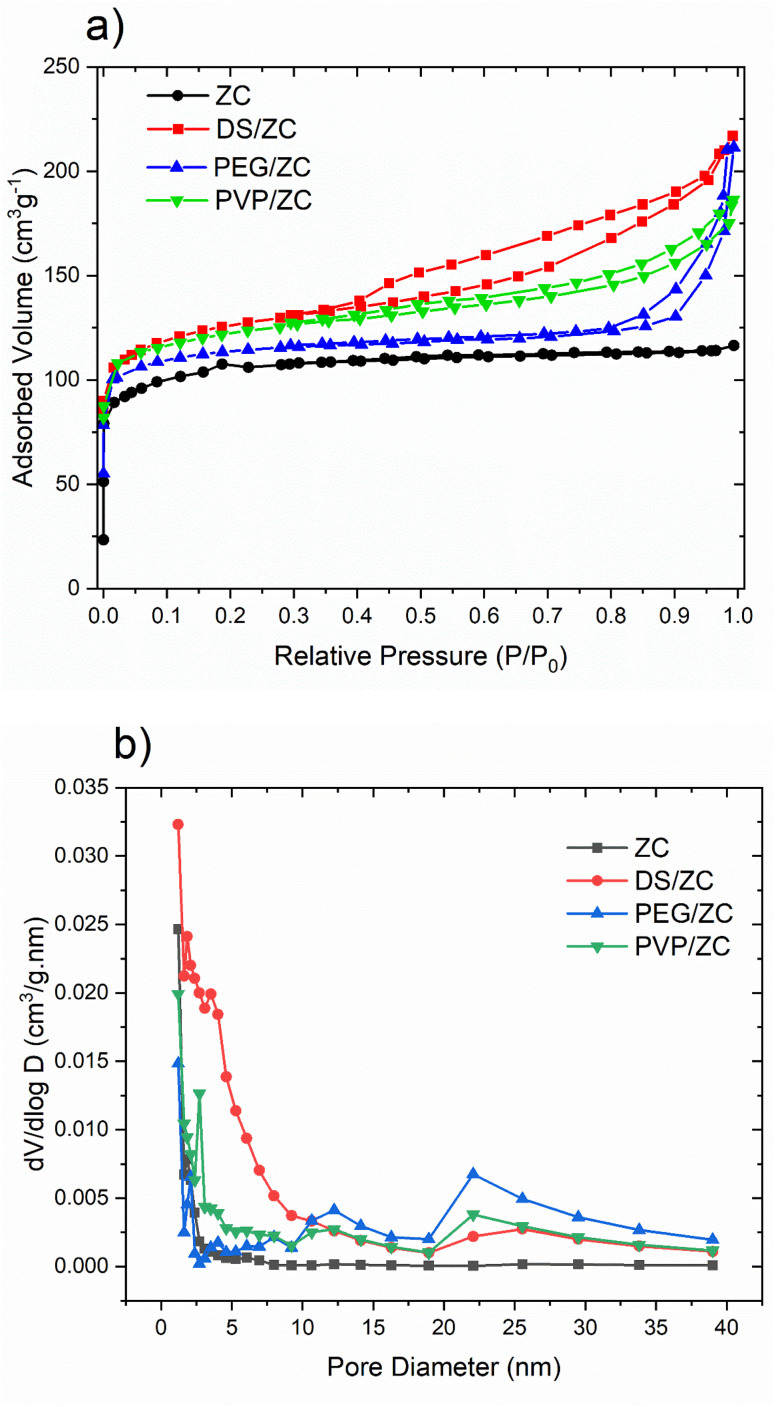
(a) N_2_ adsorption–desorption isotherms, (b) BJH pore size distribution plot of catalyst samples.

In order to study the pore structure, the catalysts were characterized by N_2_ adsorption–desorption measurement. The results are shown in Fig. 3a and Table 1. The adsorption–desorption isotherms of ZC exhibit a typical type-I isotherm characterized by adsorption at low relative pressures (*P*/*P*_0_ < 0.1) and a long plateau at higher relative pressures (0.4 < *P*/*P*_0_ < 0.9). This pattern suggests that the ZC zeolite is a purely microporous material with little mesoporosity.

**Fig. 4:**
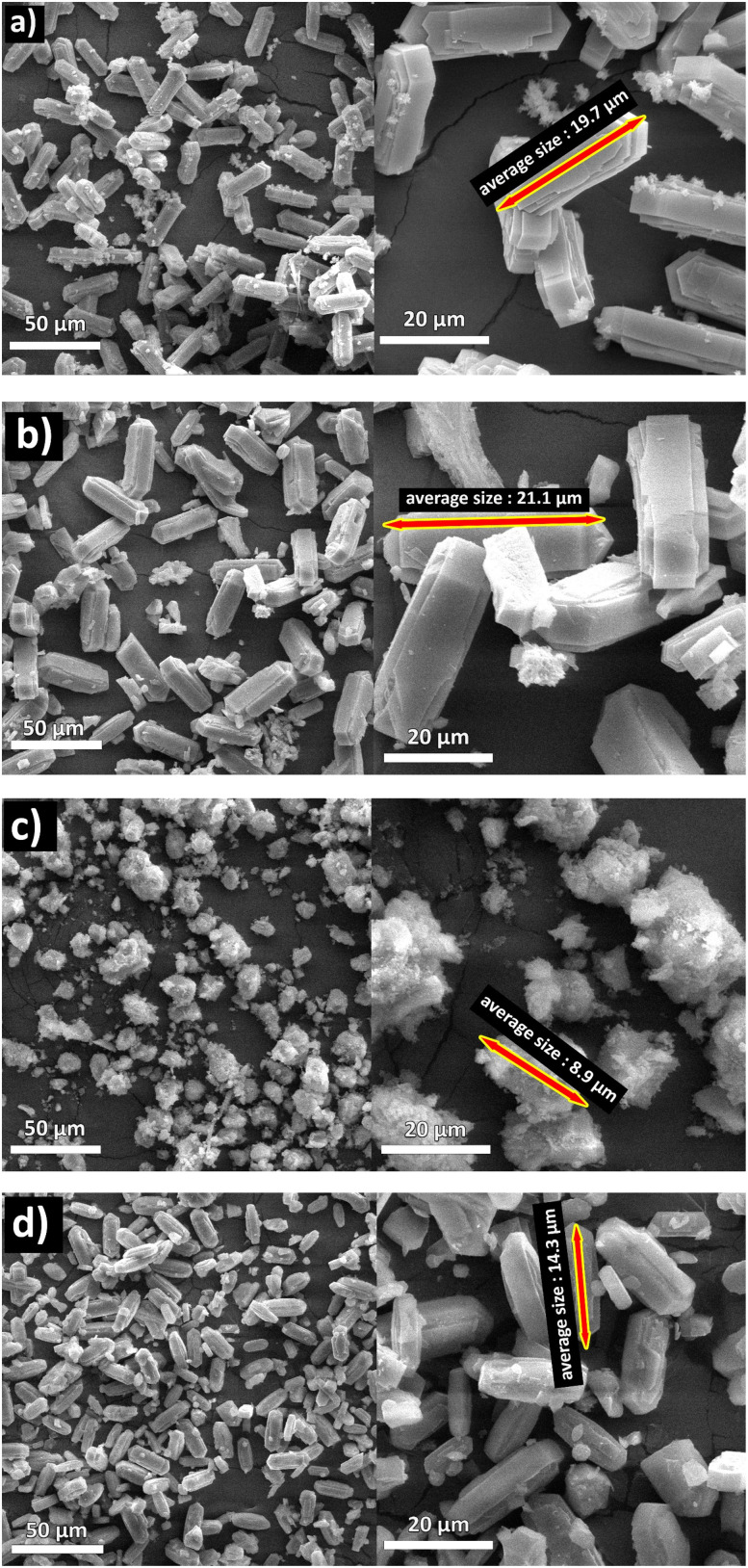
SEM images of (a) ZC lamellar hexagonal morphology, (b) DS/ZC lamellar hexagonal morphology with partial surface destruction, (c) PEG/ZC irregular ellipsoidal, and (d) PVP/ZC coffin shape morphology.

**Fig. 5:**
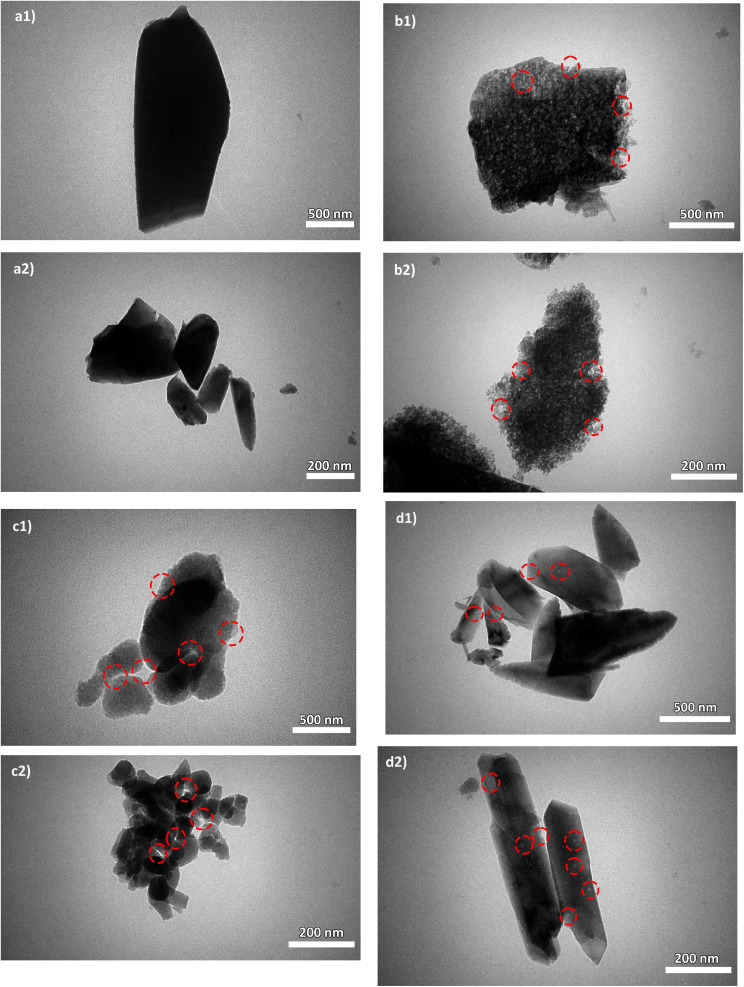
TEM images of (a1 and 2) ZC: fully microporous MFI framework without visible mesopores, (b1 and 2) DS/ZC: exhibits irregular mesopores originating from desilication, (c1 and 2) PEG/ZC: displays a combination of medium-sized mesopores and larger voids caused by incomplete crystallization, and (d1 and 2) PVP/ZC: presents uniform intracrystalline mesopores while preserving the micropore network.

The DS/ZC and PVP/ZC exhibit a type-IV isotherm with an H4 type hysteresis loop in the range of *P*/*P*_0_ = 0.4–0.99, referring to the coexistence of microporous and mesoporous, which is confirmed by SEM and TEM images (Fig. 4 and 5). Also, PEG/ZC shows the same H4 isotherm type hysteresis loop in the range of *P*/*P*_0_ = 0.8–0.99, which suggests the presence of a small amount of particle-piled macropores, which is consistent with other literature.^52^

The textural properties of the zeolite samples are summarized in Table 1. ZC specific surfaces of 308.9 m^2^ g^−1^ as well as total pore volumes of 0.179 cm^3^ g^−1^. The alkali treatment of this sample increases surface area and total volume to 373.6 m^2^ g^−1^ and 0.329 cm^3^ g^−1^. Besides, desilication rises *S*_ext_ and *V*_meso_ from 53.8 m^2^ g^−1^ and 0.014 cm^3^ g^−1^ to 90.4 m^2^ g^−1^ and 0.122 cm^3^ g^−1^ by fabricating mesoporosity. PEG/ZC indicates a total volume, and *V*_meso_ reached 0.314 cm^3^ g^−1^ and 0.252 cm^3^ g^−1^, respectively, according to the presence of PEG as mesoporogen agent. In contrast, *V*_micro_ decreases by about 62% in comparison with ZC. This might be due to the presence of high molecular weight PEG, which causes the crystallization process not to be completed, resulting in the existence of crystalline and amorphous phases in the sample, which accumulate to form intracrystalline mesopores and macropores, consistent with the SEM images and relative crystallinity from XRD data. The surface area and total volume in the PVP/ZC sample reach 359.1 m^2^ g^−1^ and 0.287 cm^3^ g^−1^, respectively. As is clear, the mesopore volume and micropore volume are 0.183 cm^3^ g^−1^ and 0.104 cm^3^ g^−1^, respectively, which shows that PVP not only creates a hierarchical mesopore structure with high crystallinity, but also preserves the intrinsic micropore properties.

The BJH pore size distribution shown in Fig. 3b shows that the ZC sample has the highest peak in the microporous region, revealing a large number of pores with a diameter of about 1–2 nm. The DS sample has a higher contribution in the mesopore region, *i.e.*, 3–6 nm. The PVP sample is marked by mesopores with a predominance in the 2–6 nm range, along with a small contribution in the 10–20 nm range. Additionally, the PEG/ZC sample shows a broader range of mesopores from 2 to 5 nm and up to 20 to 50 nm.

The DS/ZC sample develops non-uniform mesopores due to uncontrolled desilication, partially preserving its micropores but introducing significant structural defects through Si/Al leaching. In contrast, PEG/ZC shows broad and irregular mesoporosity, including macropores arising from incomplete crystallization; this morphology results in a ∼62% loss of micropore volume, weakening shape selectivity and promoting the formation of heavier C_9+_ aromatics. By comparison, PVP/ZC exhibits uniform intracrystalline mesopores while fully maintaining the intrinsic microporous network, thereby retaining shape selectivity and favoring the formation of BTX aromatics. These structural distinctions highlight the critical role of controlled mesoporosity generation in optimizing catalytic performance.

#### 3.1.4. Morphology analysis by SEM and TEM

SEM characterization reveals the morphologies of all ZSM-5 samples. All samples represented single-crystalline morphologies. As shown in Fig. 4a, ZC zeolite exhibits a lamellar hexagonal shape with a crystal size of about 19.7 µm. Zeolite DS also maintains its morphology (Fig. 4b), with the difference that the crystal size has slightly increased to 21.1 µm, and the effects of the desilication process are also observed. However, the PEG/ZC sample shows a different morphology. As depicted in Fig. 4c, it presents an irregular ellipsoid shape with an average crystal diameter of 8.9 µm; however, an amorphous phase is also visible.

Fig. 4d shows the morphology of PVP zeolite, similar to ZC zeolite, except that the crystal size has been reduced to 14.3 µm, and its shape has also become coffin-shaped. Polymer templates, due to their hydrophilic and hydrophobic properties, can interact with amorphous precursors or zeolite frameworks to control nucleation and crystal growth, thereby serving as morphology modulators. In the case of PEG, its mechanism involves both coordination and steric effects: the ether oxygens and terminal –OH groups coordinate with Al–OH/Si–OH groups on nuclei, anchoring the polymer chains to their surfaces. This interaction often decreases the number of nuclei by stabilizing the gel's homogeneity, leading to numerous small nuclei and, ultimately, smaller crystal particles during the early crystallization stages. Additionally, PEG adsorbs onto zeolite precursors, forming a barrier that limits further growth, while its chain length influences mesopore formation. At higher molecular weights, PEG tends to slow down or even halt crystallization, resulting in lower crystallinity or less ordered frameworks. PEG also disrupts the Ostwald ripening process. In conventional ZSM-5 synthesis, small nuclei dissolve and redeposit on larger crystals, leading to well-faceted particles. PEG adsorbs strongly onto smaller proto-crystals, sterically protecting them against dissolution. This inhibition of ripening lead to populations of small, irregular crystals and promotes the formation of intercrystalline voids that act as mesopores. It has been reported that such ‘growth arrest’ effects significantly reduce the order of micropore and increase the number of defect–rich interfaces, consistent with the morphology observed in the PEG/ZC sample.^60^

In contrast, PVP interacts through its pyrrolidone units, where the lactam carbonyl (–C=O) and tertiary amine groups can hydrogen-bond or coordinate with Al–OH/Si–OH groups on nuclei or crystals. This interaction, combined with the steric hindrance from the polymer chains, significantly inhibits crystal growth and reduces the size of zeolite particles. Furthermore, the permanent dipole moment of the carbonyl group slightly enhances ionic mobility in solution, solubilizes cations, and prevents their aggregation, acting as a carrier to maintain ionic species' accessibility during crystallization. It seems that PVP can influence the distribution of framework Al by selectively stabilizing tetrahedral Al species during the early stages of hydrothermal treatment. The strong dipole moment of the lactam carbonyl group interacts preferentially with partially charged AlO_4_^−^ units, preventing their migration and reducing the formation of extra-framework Al. This templating-like effect helps preserve microporosity and generates a more uniform distribution of acid sites.^61^ Unlike PVP, PEG is neutral and nonionic, so its electrostatic contribution is much weaker, but it mainly affects the framework structure through steric and chain-length effects.^54,55,61–64^

Furthermore, HZSM-5 features two sets of intersecting channels with comparable opening sizes but distinct shapes. The 0.51 × 0.55 nm sinusoidal channels are smaller and more convoluted compared to the straighter 0.52 × 0.58 nm channels, as reported in the literature.^46,62^ Thus, straight channels along the *b*-axis are more favorable for aromatic selectivity. Consequently, the *b*-axis and *c*-axis dimensions of the catalyst are determined and presented in Table 2. As shown in Table 2, the length along the *b*-axis of the catalyst follows the trend PVP/ZC < ZC < PEG/ZC and DS/ZC.

**Table 2:** The average size of the prepared catalyst

Catalyst	Average size (µm)	Size along *b*-axis (µm)
ZC	19.7	4.4
DS/ZC	21.1	6.2
PEG/ZC	8.9	5.4
PVP/ZC	14.3	2.0

TEM characterization was carried out as a complement to SEM images to provide further insight into the morphology and hierarchical structure of ZSM-5. As shown in Fig. 5a1 and a2, the ZC catalyst exhibits a hexagonal morphology; no mesoporosity could be observed from the crystals. After alkaline treatment, intracrystalline mesoporosity was present (Fig. 5b1 and b2). To assist visual interpretation, representative mesoporous regions are highlighted in the TEM images. Also, Fig. 5c1, c2, d1 and d2 show that the TEM images of PEG/ZC and PVP/ZC exhibit intracrystalline mesopores, as demonstrated by the whiter tonality in the zeolite crystalline frameworks, which is consistent with the BET analysis. PVP/ZC has a narrower, uniform, mesoporous structure; in contrast, PEG/ZC shows broader mesoporosity distribution, and larger mesopores (even macropores) are visible.

In brief, SEM and TEM morphological analysis emphasized that the pore and crystal structures of the synthesized samples differ markedly, leading to different diffusion pathways and coking behaviors. PEG/ZC consists of irregular ellipsoidal particles (∼8.9 µm) with amorphous regions, indicating the disruptive influence of PEG on crystal growth and resulting in heterogeneous mesopores and large voids. Conversely, PVP/ZC forms well-defined coffin-shaped crystals (∼14.3 µm) with smooth surfaces, and TEM confirms uniform intracrystalline mesopores formed through controlled nucleation and growth facilitated by PVP. The DS/ZC maintains much of the original ZC morphology but exhibits surface roughness, etching marks, and irregular mesopores formed by nonselective Si extraction during alkaline desilication. Meanwhile, the original ZC features large lamellar crystals (∼19.7 µm) with no apparent mesoporosity. Despite all catalysts sharing the MFI framework, their particle shapes, defect levels, and mesopore structures are fundamentally different, indicating they should be regarded as distinct catalytic materials. These morphological differences influence the accessibility of the active-site, the diffusion of aromatic intermediates, and ultimately, their catalytic performance in the MTA process.

#### 3.1.5. NH_3_-TPD analysis

**Fig. 6:**
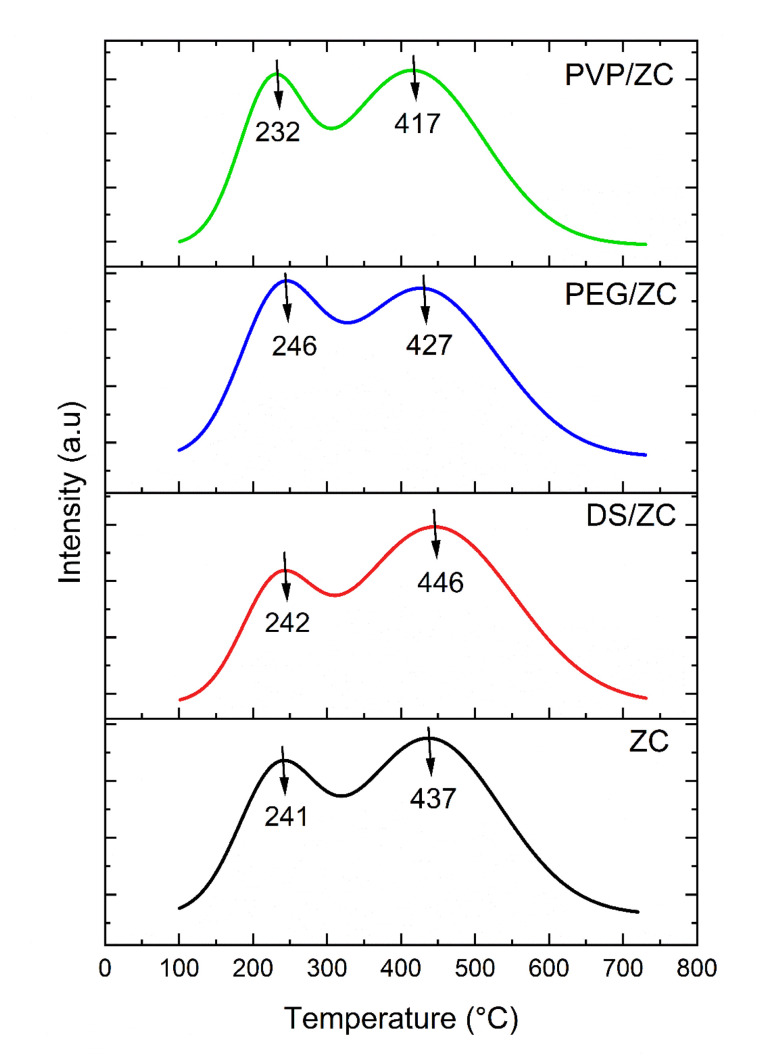
NH_3_-TPD profiles of ZC, DS/ZC, PEGLZC, and PVP/ZC.

NH_3_-TPD deconvolution separates overlapping desorption peaks, allowing clear identification of acid sites with varying strengths. Following baseline correction, the TPD curve is modeled using Gaussian functions, where the position of each peak represents the acid strength and its area indicating the amount of ammonia released. This approach provides a more accurate measurement of weak and strong acidity than the raw TPD data profile, as shown in Fig. 6. Based on the desorption temperatures, the corresponding acid amount is summarized in Table 3. Temperature range of 230–250 °C and above 420–450 °C, which belong to the desorption of physisorbed NH_3_ species from the weak acid site (possibly Lewis acid sites) and stronger acid site (possibly Brønsted acid sites), respectively.^65^ Based on the literature,^66,67^ the weak acid sites result from the Al species of extra- and non-framework (Si–OH–Al in structural defects). In contrast, strong acid sites are often introduced by the framework Al. The parent ZC sample exhibits a total acidity of 0.680 mmol g^−1^, dominated by strong acid sites (0.503 mmol g^−1^). DS/ZC shows a decrease in total acidity to 0.617 mmol g^−1^, accompanied by a reduction in strong acidity from 0.503 to 0.470 mmol g^−1^, indicating partial dealumination of framework Al, consistent with ICP results.^68^ Literature suggests that the alkali treatment of low Si/Al ZSM-5 might damage not only Si, but also the framework Al, based on uncontrollable desilication.^69,70^ PEG/ZC shows the highest acidity increase to 0.280 mmol g^−1^ and 0.881 mmol g^−1^ for weak and strong acidity, corresponding to the lowest Si/Al ratio. This behavior can be attributed to the role of PEG, whose multiple polar –OH groups interact with Al^3+^ species in the synthesis gel, maintaining their dispersion and gradually releasing them during crystallization. Such a carrier-like effect facilitates framework Al incorporation and increases strong acid site density.^52^ Also, PVP/ZC shows more acidity compared to the ZC sample. Considering that in this sample, weak acidity has increased significantly from 0.176 mmol g^−1^ to 0.291 mmol g^−1^, in addition to the increase in strong acidity. PVP is created by creating mesopores, which enhances the distribution of acid sites and their accessibility,^58,71^ and can also influence the distribution of acid sites, allowing for tuning the ratio of strong to weak acid sites,^53^ Moreover, the stronger interaction between PVP and Al species, along with potential changes in gel viscosity, may hinder the less incorporation of Al into the zeolite lattice compared to PEG. Consequently, fewer framework strong sites are formed (0.449 mmol g^−1^*vs.* 0.601 mmol g^−1^), while isolated silanol groups and extra-framework Al species can create weaker acid sites (0.291 mmol g^−1^*vs.* 0.280 mmol g^−1^). This explains why PVP/ZC shows higher weak acidity and lower strong acidity than PEG/ZC, as reported in Table 3, which influences MTA reaction performance.

**Table 3:** Acidic properties of prepared catalysts

Catalyst	Weak acidity (mmol g^−1^)	Strong acidity (mmol g^−1^)	Total acidity (mmol g^−1^)	Strong/weak acid ratio
ZC	0.176	0.503	0.680	2.8
DS/ZC	0.147	0.470	0.617	2.9
PEG/ZC	0.280	0.601	0.881	2.1
PVP/ZC	0.291	0.449	0.740	1.5

According to NH_3_-TPD data, the varying acidity levels of samples tend to direct each catalyst toward different reaction pathways in the MTA process. PEG/ZC shows the highest strong acidity (0.601 mmol g^−1^), which promotes hydrogen-transfer reactions and encourages the formation of heavy aromatics. Conversely, PVP/ZC offers the highest concentration of weak acid sites along with a well-balanced strong/weak acidity ratio, a pattern widely recognized as ideal for BTX production. The DS/ZC sample demonstrates a significant reduction in total acidity due to partial framework dealumination during desilication, while the parent ZC retains its original microporous structure with predominantly strong acid sites. In general, these acidity profiles determine the respective roles of oligomerization, dehydrogenation, and cracking pathways in the catalysts.

### 3.2. Catalytic performance

ZSM-5 zeolites are widely used for the conversion of methanol to hydrocarbons due to their physicochemical properties. In our study, all the synthesized ZSM-5 catalysts are used to evaluate their catalytic performance in the aromatization of methanol, and the product selectivity results at 3 h time on stream (TOS) are shown in Table 4. Methanol conversion remains above 99.5% throughout the reaction.

**Table 4:** Product selectivity for MTA reaction over the ZSM-5 samples^a^

Catalyst	Selectivity (%)									
	C_1_	C_2_–C_4_	C_2_–C_4_=	C_5+_	B	T	X	C_9+_	Aromatic	BTX
ZC	1.9	45.7	8.5	6.5	6.9	7.3	9.8	13.2	37.2	24
DS-ZC	2.1	28.5	14.1	10.8	3.7	9.1	15.3	16.4	44.5	28.1
PEG-ZC	3.1	26.3	3.4	4	5.3	10.8	16.6	30.5	63.2	32.7
PVP-ZC	2.5	25.0	6.3	6.1	6.2	12.6	32.4	8.8	60.1	51.3

a Reaction conditions: TOS = 3 h, atmospheric pressure, 0.6 g catalyst, 400 °C, WHSV = 5 h^−1^. The conversion of methanol is 99.5% for all samples. C_1_: methane, C_2_–C_4_ alkanes hydrocarbons, C_2_–C_4_= olefin hydrocarbons, C_5+_ aliphatic hydrocarbons, B: benzene, T: toluene, X: xylenes, C_9+_: heavy C_9+_ aromatic hydrocarbon.

Methanol conversion to hydrocarbons is a complicated chemical process that relies on the morphological, acidic, and structural characteristics of the zeolite. Methanol molecules generally move to the catalyst's surface and are transformed into primary products at acid sites. Subsequently, these primary products penetrate the interior of the catalyst and go through a sequence of tandem reactions, including dehydration, methylation, oligomerization, dehydrogenation, hydrogen transfer, and cyclization, to form aromatics.

Additionally, other reactions, such as alkylation and cracking, may produce undesirable products. The conventional ZSM-5 zeolite, ZC, has an aromatic selectivity of 37.2% and a BTX selectivity of 24%. The selectivity of C_2_–C_4_ alkanes, on the other hand, is the main product, with 47.5% selectivity. The high selectivity for light alkanes shows that the reaction mostly goes through cracking pathways, which makes smaller hydrocarbon fragments more likely to form.

This behavior is characteristic of ZSM-5's strong acidity and its microporous structure, which facilitates β-scission reactions that lead to the formation of light alkanes. However, the microporous nature of ZSM-5 can impose diffusion limitations, particularly for larger intermediates and products, which may hinder further transformation into aromatics. Alkali-treated sample, DS/ZC, shows a better aromatic and BTX selectivity, rising to 44.5% and 28.1%. In this sample, *S*_BET_ and total volume up to 373.6 m^2^ g^−1^ from 308.9 m^2^ g^−1^ and 0.329 cm^3^ g^−1^ from 0.179 cm^3^ g^−1^, and this mesoporosity formation is valuable in enhancing molecular diffusivity, which allows reactant molecules easier access to active sites in the micropores, leading to better aromatic selectivity. Nevertheless, the unruly mesoporous structure and intense damage to the structure by harsh NaOH leaching are promising for carbon chain growth, which caused more selectivity to C_9+_ hydrocarbons compared to ZC. On the other hand, non-uniform desilication resulted in the leaching of Si and Al, as confirmed by ICP analysis. This process led to a reduction in both weak and strong acidity, as indicated by the data in Table 3 (refer to NH_3_-TPD). The decrease in acidity has caused a decline in alkane production while simultaneously increasing C_2_–C_4_= olefin and C_5+_ aliphatic production. Notably, there has not been a significant increase in BTX production. The PEG/ZC catalyst exhibits the highest aromatic selectivity, at 63.2%, but also shows a BTX selectivity increase of only 8.7% compared to ZC, reaching 32.7%. PEG/ZC has the highest strong acid concentration (0.601 mmol g^−1^), corresponding to the lowest Si/Al ratio. According to the literature, oligomerization, cracking, and hydrogen transfer reactions mainly take place on strong acid sites.^72^ A high number of these sites can promote oligomerization and hydrogen transfer, thereby increasing the selectivity of the aromatics. Besides, PEG/ZC has the largest *S*_ext_ and *V*_meso_ and very low *V*_micro_. The partial destruction of channels and pore openings negatively impacts BTX selectivity. This is due to the loss of shape-selective pores and the emergence of newly formed, non-shape-selective acid sites, which promote the growth of aromatic intermediates and lead to the production of heavy hydrocarbons (C_9+_). PVP/ZC not only has high aromatic selectivity (60.1%), but also shows the most considerable BTX selectivity, reaching 51.3%. According to the TEM analysis, PVP/ZC exhibits uniform mesoporosity while preserving its micropore structure, with a *V*_micro_ of 0.104 cm^3^ g^−1^ and a *V*_meso_ of 0.183 cm^3^ g^−1^. In contrast to the PEG catalyst, PVP/ZC demonstrates good shape selectivity and effectively overcomes mass transfer limitations in the formation of BTX aromatics, thanks to its mesopore structure. Diffusion of aromatic compounds in ZSM-5's three-dimensional channel system (two intersecting channels) favors the straight channels. Conversely, small molecules such as ethylene and propylene permeate both straight and sinusoidal channels. As indicated in Table 2, PVP/ZC has the shortest straight channel among the catalysts listed, which enhances the diffusion of the produced aromatics and results in higher selectivity for aromatics. Additionally, the efficient diffusion of aromatics reduces the likelihood of secondary reactions, such as alkylation and polymerization of the aromatics, thereby inhibiting the formation of heavier hydrocarbons. As a result, the selectivity for C_9+_ hydrocarbons decreased to 8.8% with the PVP/ZC catalyst, compared to 30.5% with the PEG/ZC catalyst. On the other hand, the PVP/ZC catalyst exhibits a higher acidity compared to the ZC and DS/ZC catalysts, which consequently enhances its selectivity towards aromatics. In contrast to the PEG/ZC catalyst, the PVP catalyst demonstrates a predominance of weak acidity over strong acidity. This shift in acidity profile facilitates the dehydrogenation reaction, thereby increasing selectivity for BTX while concurrently suppressing the formation of heavy hydrocarbons and alkanes. This result demonstrates the remarkable catalytic performance in terms of BTX selectivity affected by the synergy between acidity and porosity, and the type of mesopore template is conclusive.

PVP/ZC, with its balanced acidity and relatively higher fraction of weak acid sites, promotes controlled methylation and dehydrogenation pathways that preferentially yield BTX molecules. However, PEG/ZC contains the highest concentration of strong Brønsted sites, accelerating hydrogen-transfer and oligomerization reactions that divert the products toward heavier aromatics and coke precursors. The lower acidity of DS/ZC arising from partial dealumination limits its capacity for efficient aromatization, resulting in only little improvement relative to the parent ZC.

The PVP/ZC catalyst developed in this study was compared with other catalysts reported in the literature for the MTA reaction, as summarized in Table 5. Results indicate that the PVP/ZC catalyst significantly enhances BTX selectivity in the MTA process.

**Table 5:** Comparison of catalyst samples with literature reports for MTA

Catalyst	Synthesis method	Reaction condition *T* (°C), *P* (bar), TOS (h)	BTX selectivity (%)	Aromatic selectivity (%)	BTX/Aromatic ratio (%)	Ref.
e-H-ZSM-5	One-pot hierarchical ZSM-5 synthesis with a tuned precursor composition and 72 h of synthesis time, at 170 °C, TPAOH as the major ZSM-5 template	450, 5, 2	38	42	90.5	16
NZ-2000	One-pot hierarchical ZSM-5 synthesis with a tuned H2O/Al ratio and 48 h of synthesis time, at 170 °C, TPAOH as a major ZSM-5 template	400, 1, 11.5	39.6	66	60.0	39
BZ5	Synthesis with ageing precursor solution, 48 h of synthesis time, at 170 °C, TPAOH as the major ZSM-5 template	430, 1, 3	24.2	40.6	59.6	8
NSHZ	One-pot synthesis with a mesoporgen agent, 72 h of synthesis time, at 170 °C, TPAOH as the first ZSM-5 template, and KH-560 as the second template	400, 1, 10	36.5	47.6	76.7	22
NZ3	One-pot synthesis with a mesoporgen agent, 72 h of synthesis time, at 170 °C, TPAOH as the first ZSM-5 template, and KH-560 as the second template	400, 1, 6	48.2	62.6	77.0	78
Z5/F127E60	Synthesis with ageing precursor solution, with a mesoporgen agent, 42 h of synthesis time at 170 °C, TPAOH as the first ZSM-5 template, and F127 as the second template, ethanol as co-solvent	450, 1, 3	65.8	71.9	91.5	53
Z5-Na + TP	Desilication of commercial ZSM-5 with mixed alkali solution (NaOH + TPAOH) for 4 h, at 80 °C and following surface sialylation	400, 1, 3	39.5	69.4	56.9	24
H-ZSM-A	Synthesis of micropore ZSM-5 with a 72 h synthesis time, at 170 °C, followed by hierarchical ZSM-5 synthesis with desilication using NaOH solution for 24 h, at 170 °C	450, 1, 6	59.5	62.9	94.5	72
PVP/ZC	One-pot synthesis with a mesoporgen agent, 48 h of synthesis time, at 170 °C, NBA as the first ZSM-5 template, and PVP as the second template	400, 1, 3	51.3	60.1	85.4	This work

**Fig. 7:**
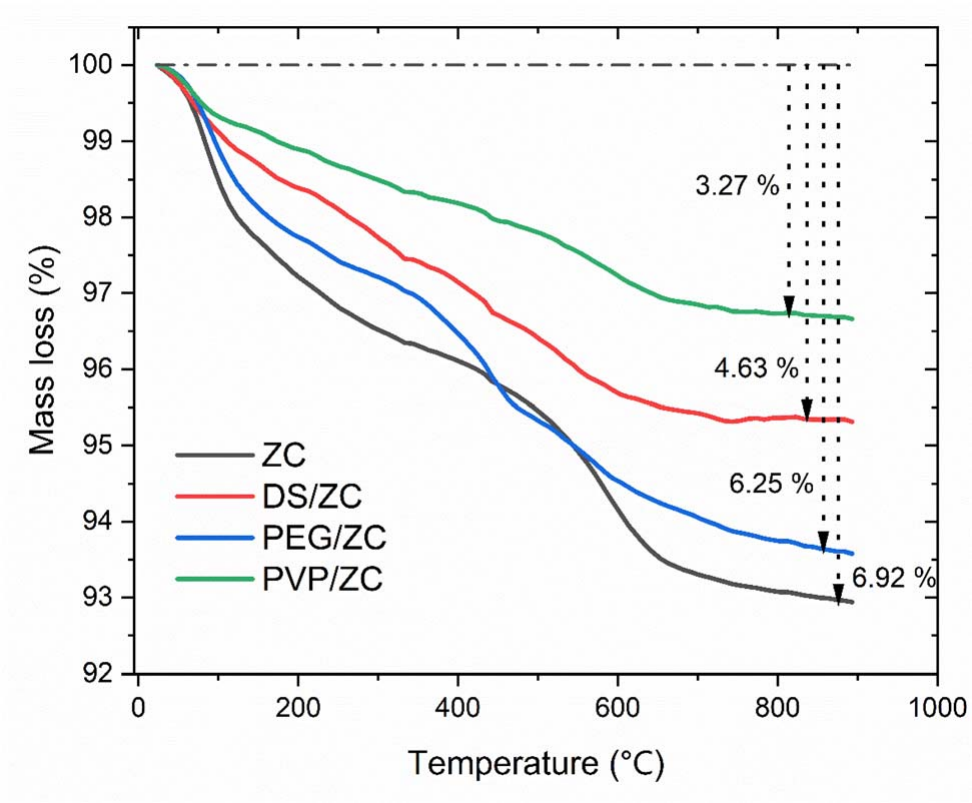
TG profiles of the catalysts after the MTA reaction.

**Fig. 8:**
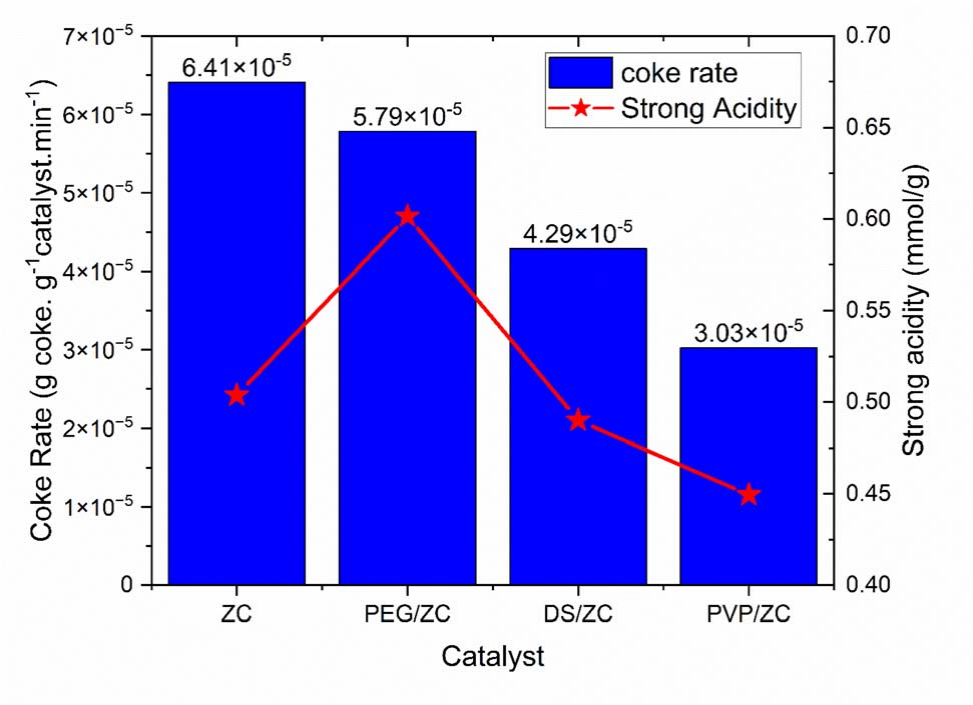
Coke rate production of ZSM-5 samples.

The carbon deposition on the catalysts after the MTA reaction was studied using the thermogravimetric (TG) technique. Fig. 7 presents the TG profiles of all catalysts following MTA reactions for 3 hours. These curves exhibited two stages of weight loss: one occurring below 300 °C, attributed to water desorption, and another at temperatures ranging from 300 to 800 °C, which was attributed to coke decomposition, respectively. Calculations revealed that the weight loss was 6.92%, 6.25%, 4.63%, and 3.27% for ZC, PEG/ZC, DS/ZC, and PVP/ZC catalysts, respectively. Meanwhile, Fig. 8 displays the generation rate of coke was 6.4 × 10^−5^ g g^−1^ min^−1^, 5.7 × 10^−5^ g g^−1^ min^−1^, 4.2 × 10^−5^ g g^−1^ min^−1^, and 3.0 × 10^−5^ g g^−1^ min^−1^ for ZC, PEG/ZC, DS/ZC, and PVP/ZC catalysts within 3 h of the reaction time, respectively. The ZC catalyst generates and accumulates the highest amount of coke due to its micropore structure, despite having the lowest surface area and pore volume. Interestingly, the PEG/ZC, which possesses the largest external surface area, has the highest coke generation rate among the hierarchical catalysts. As shown in Fig. 2b, external silanol groups contribute to an increased coke rate.^17,73^ Additionally, Fig. 8 indicates that the presence of strong acids in hierarchical catalysts correlates with coke production rate; specifically, a greater amount of strong acidity leads to a higher coke production rate. Consequently, the PVP/ZC catalyst exhibits the lowest rate of coke production due to the lowest strong acidity. This relationship can be explained mechanistically: strong acid sites speed up key steps such as oligomerization, hydrogen transfer, and over-methylation of hydrocarbon intermediates, leading to the formation of heavier oligomeric or polyaromatic species that are coke precursors. These heavier intermediates are more prone to undergo polymerization and condensation, especially near or at external-surface sites and in silanol-rich regions where shape selectivity is lost and mass transport encourages accumulation. Therefore, catalysts with higher strong acidity promote faster coke nucleation and growth. Conversely, catalysts that maintain micropore shape selectivity and have moderate to strong/weak acid balance facilitate faster removal of aromatics from active sites, suppress over-methylation and oligomerization, and consequently lead to lower coke production rates.^17,74^

**Fig. 9:**
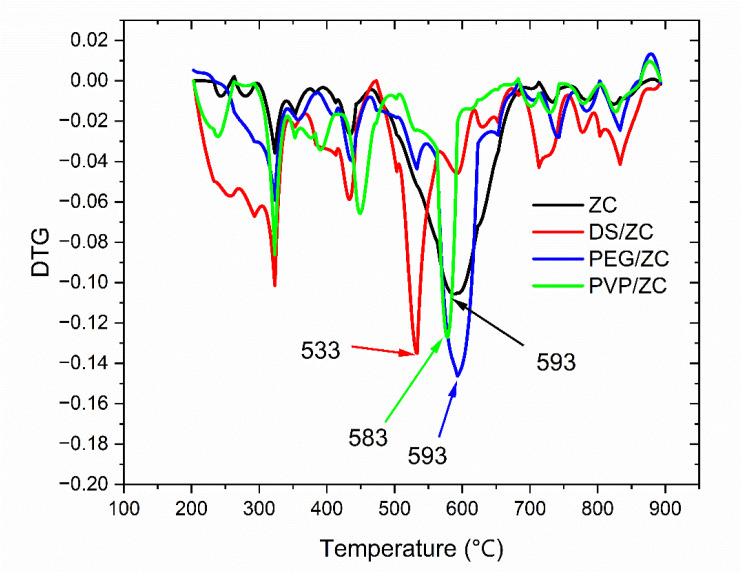
DTG profiles of the catalysts after the MTA reaction.

As shown in the Fig. 9, the DTG profiles exhibit two distinct peaks in which the first step, between 200 and 480 °C, involves the removal of oxygenates and light hydrocarbons, commonly known as soft coke and having a relatively high H/C ratio. The second, between 480 and 650 °C, corresponds to polycondensed aromatic coke, representing hard coke with a lower H/C ratio.^75–77^ It is noteworthy that the PVP-derived sample contains a larger amount of soft coke compared to the DS/ZC and PEG/ZC catalysts, which tend to accumulate more highly condensed hard coke. Moreover, the high-temperature DTG peaks (>480 °C) for PEG/ZC and ZC appear around 593 °C, while those for PVP/ZC and DS/ZC shift to lower temperatures, suggesting that coke on PEG/ZC and ZC is more highly condensed.^10^ This aligns with their stronger acidity and predominantly microporous structures, respectively.

## 4. Conclusion

The present investigation focuses on the synthesis of hierarchical ZSM-5 catalysts with superior methanol-to-aromatic performance. The major conclusions are summarized as:

(1) One-pot synthesis of hierarchical ZSM-5 with eco-environment-friendly templates with novel composition and investigation of their physico–chemical properties compared to the desilicated hierarchical sample shows that a suitable mesoporogen could affect remarkable properties on ZSM-5. Also, the composition and condition of synthesis shall be more tuned for further studies.

(2) The desilicated sample, DS/ZC, shows larger surface area and pore volume, but also due to the uneven desilication and leaching of Si and Al of the framework, causing a reduction in acidity of the catalyst, that have a bad effect on aromatic and BTX selectivity.

(3) The use of water-soluble polymers has created zeolites with hierarchical properties suitable for the MTA process. The PEG/ZC zeolite, with an ellipsoidal shape, reduces particle size by around 54% and enhances external surface area and pore volume. It also has the highest acidity among the samples. However, due to high molecular weight PEG, crystallization was incomplete, resulting in the lowest RC. The zeolite exhibits a notable aromatic selectivity of 63.2%, but its BTX selectivity is suboptimal, producing a significant amount of C_9+_ hydrocarbons, likely due to its large external pore surface and strong acidity. Additionally, the PEG catalyst has the highest coke production rate among the hierarchical samples.

(4) The addition of PVP in zeolite synthesis improves its properties. Morphological analysis reveals that the PVP catalyst exhibits a Coffin-shaped structure with regular mesopores, while maintaining a microporous structure. PVP also promotes smaller zeolite particle sizes, enhancing aromatic selectivity by reducing the *b*-axis size. Although PVP/ZC has lower strong acidity than PEG/ZC, its weak acidity has increased, minimizing heavy hydrocarbon production and improving BTX selectivity. As a result, PVP/ZC exhibits the highest selectivity for BTX.

(5) This study highlights the critical role of water-soluble polymer templates in tailoring the textural and acidic properties of ZSM-5. It demonstrates how structural modifications directly correlate with product selectivity in the MTA reaction. The results underline the potential of PVP-templated hierarchical ZSM-5 as a promising candidate for industrial applications, offering enhanced BTX yield and improved resistance to deactivation. Future studies will focus on long-term stability, regeneration behavior, and techno–economic assessment to further validate the industrial applicability of this synthesis strategy.

## Conflicts of interest

There are no conflicts to declare.

## Data Availability

The authors declare that they have no known competing financial interests or personal relationships that could have appeared to influence the work reported in this paper. All experimental data are presented in this article.
